# A Case of Congenital Obstruction of the Bile-Duct

**Published:** 1931

**Authors:** J. A. Birrell, A. Wilfrid Adams

**Affiliations:** Assistant Physician, Bristol General Hospital; In-patient Physician, Children's Hospital, Bristol; Senior Assistant Surgeon, Bristol Royal Infirmary; Senior Surgeon with charge of Out-patients, Children's Hospital, Bristol


### A CASE OF CONGENITAL OBSTRUCTION OF THE BILE-DUCT.

BY

J. A. Birrell, M.D., M.R.C.P.,

*Assistant Physician, Bristol General Hospital ; In-patient Physician, Children's Hospital, Bristol.*

AND

A. Wilfrid Adams, M.S., F.R.C.S.,

*Senior Assistant Surgeon, Bristol Royal Infirmary ; Senior Surgeon with charge of Out-patients, Children's Hospital, Bristol.*

---

The following case, recently under our observation at the Children's Hospital, presented features which deserve report.

*Diagnosis and Clinical Course.*—Dr. Milling, to whom we are indebted for the opportunity of observing this baby, told us that the labour was normal. There were no convulsions, rashes or snuffles. The weight at birth was 8½ lb. The usual post-natal icterus did not, a few days after birth, entirely disappear. It was thenceforward replaced by a jaundice becoming deeper in tint, and an enlargement of the liver presented itself.

On admission to hospital, at the age of 4 months, the child weighed 10 lb. 12 oz. She appeared comfortable, in a fair state of nutrition. She was moderately jaundiced to a greenish-yellow hue. She took her feeds of ordinary diluted citrated milk well, without abnormal vomiting. There were two or three motions a day. The stools were absolutely white, of a dried powdery curd consistency. The upper abdomen was prominent ; the liver easily palpable, firm, rather finely nodular, with a rough, well-marked edge at the umbilical level. The splenic tip could just be felt.

Clinically, congenital obstruction of the bile-duct seemed the only and obvious diagnosis. There were no features of congenital syphilis, and the Wassermann reaction, though perhaps somewhat unreliable at this age, was negative. There was no reason to believe there was cystic or neoplastic pressure on the bile-duct from without ; there was no obvious irregularity of the liver surface ; calculous or parasitic obstruction of the bile-duct seemed unlikely. The jaundice was more intense than one expects in congenital acholuric icterus ; moreover, the hepatic enlargement preponderated over that of the spleen, whereas the reverse is the case in this disease. The Van den Bergh reaction was positive, direct and indirect.

As time went on the jaundice became more marked during the ensuing five weeks, varying in intensity as is usual in these cases. There were remissions in the enlargement of the liver, but in the long run its size increased. Ultimately the lower edge reached the level of the iliac spine. During this time the spleen became more easily palpable. There were, however, no corresponding alterations in the stools, which retained their unblemished whiteness. The abdominal circumference over the liver was $18\frac{3}{4}$ inches, at the umbilicus $16\frac{3}{4}$ inches. (Normally it is about 14 inches in a child of this weight.)

There was a loss of 8 oz. in weight. Nevertheless, the remarkable features appeared to be the absence of constipation and vomiting, the drowsy comfort and vitality of the child. As is usual in deeply-jaundiced infants, there was not the itching or bradycardia so common in icteric adults.

The general condition was still fairly good, but slowly and steadily deteriorating. There were no cutaneous or gastric hæmorrhages.

The prolongation of a fairly intense jaundice in an infant, with the persistent absence of features suggesting any other cause of obstruction, appeared rather to confirm its congenital ætiology. Moreover, a simple catarrhal jaundice should have passed off in a week or so. This being so, it seemed appropriate to consider whether it was possible surgically to short-circuit the obstruction.

*Operation.*—At laparotomy the large liver was found very firm, lacking in surface smoothness and of greenish-black hue. Beneath it, in the normal situation, was a large, sessile, thickened gall-bladder. This tended to be sacculated towards the neck, where abnormality of the ducts was apparent. The fixity of the liver and the weak state of the infant prevented any search for the ducts.

A miniature cholecystgastrostomy was attempted and found to be just feasible. The chief hindrances were the leathery state of the walls of the gall-bladder and the fixity and downward projection of the liver. The patient rallied from the operation, but sank gradually and died a few days later.

*Post-mortem Examination.*—The examination showed an effective anastomosis through which a catheter passed easily. There was no escape of bile and no peritonitis. The biliary ducts were found to be dilated and remarkably tortuous, so that it was impossible to define exactly the representatives of the hepatic, cystic and common bile-ducts. There was no communication with the duodenum. The pancreas and other organs appeared normal.

*Remarks.*—Where congenital jaundice is attributed to stenosis of the ducts, operation affords the only hope of succour. Once the infant's hold on life is satisfactorily established then the progress of its nutrition determines the time for surgical intervention. This should take place as soon as the weight and general health reach a stationary point. To defer it means physical deterioration from increasing biliary back-pressure and greater technical difficulty owing to intensified cirrhosis.

*Conclusion.* — We consider a short-circuiting operation at the age of election, about 2 to 3 months, is feasible and affords a reasonable hope of a successful issue. There is, moreover, no alternative way of avoiding deterioration of health and early death.

